# Starch Properties and Morphology of Eight Floury Endosperm Mutants in Rice

**DOI:** 10.3390/plants12203541

**Published:** 2023-10-12

**Authors:** Yuanyuan Hao, Fudeng Huang, Zhennan Gao, Junfeng Xu, Ying Zhu, Chunshou Li

**Affiliations:** 1 Institute of Crop and Nuclear Technology Utilization, Zhejiang Academy of Agricultural Sciences, Hangzhou 310021, China; haoyuanyuan@zaas.ac.cn (Y.H.); pahfd@126.com (F.H.); gaozhennanzaas@163.com (Z.G.); 2 Key Laboratory of Traceability for Agricultural Genetically Modified Organisms, Ministry of Agriculture and Rural Affairs, Hangzhou 310021, China; njjfxu@163.com; 3 State Key Laboratory for Managing Biotic and Chemical Threats to the Quality and Safety of Agro-Products, Hangzhou 310021, China

**Keywords:** floury endosperm mutants, starch properties, starch morphology, cooking and eating quality, rice

## Abstract

Besides increasing grain yield, improving rice (*Oryza sativa* L.) quality has been paid more and more attention recently. Cooking and eating quality (CEQ) is an important indicator of rice quality. Since CEQs are quantitative traits and challenging for measurement, efforts have mainly focused on two major genes, *Wx* and *SSIIa*. Chalkiness and floury endosperm significantly affect the eating quality of rice, leading to noticeable changes in CEQ. Due to the easily observable phenotype of floury endosperm, cloning single gene mutations that cause floury endosperm and evaluating changes in CEQs indirectly facilitate the exploration of the minor genes controlling CEQ. In this study, eight mutants with different degrees of floury endosperm, generated through ethylmethane sulfonate (EMS) mutagenesis, were analyzed. These mutants exhibited wide variation in starch morphology and CEQs. Particularly, the *z2* mutant showed spherical starch granules significantly increased rapid visco analyzer (RVA) indexes and urea swelling, while the *z4* mutant displayed extremely sharp starch granules and significantly decreased RVA indexes and urea swelling compared to the wild type. Additionally, these mutants still maintained correlations with certain RVA profiles, suggesting that the genes *PUL*, which affect these indexes, may not undergo mutation. Cloning these mutated genes in the future, especially in *z2* and *z4*, will enhance the genetic network of rice eating quality and hold significant importance for molecular marker-assisted breeding to improve rice quality.

## 1. Introduction

Rice is one of the most important staple foods for more than half of the world’s population. With economic development, people not only focus on rice yield but also pay more attention to rice quality. Rice quality includes appearance quality, cooking and eating quality (CEQ), milling quality, and nutritional quality. Among them, appearance quality and CEQ have been particularly emphasized by breeders.

Appearance quality of rice includes grain shape, chalky rate, and chalkiness degree. Among them, the chalkiness degree has the most significant impact on rice quality, as it not only affects the appearance but also reduces milling yield after processing [1]. Chalkiness formation is a complex molecular and physiological mechanism involved in source-sink balance. Depending on where the chalkiness appears, chalkiness in the grain was categorized as white back, white basal, white belly, white core, and white milky [2]. Currently, more than 100 QTLs related to the degree of endosperm chalkiness and percentage of grains with chalkiness have been detected; only two, *Chalk5* and *WCR1*, have been cloned so far [3,4]. The floury endosperm is an extreme phenotype of chalkiness. Up to now, lots of genes have been identified to be involved in floury endosperm using reverse genetics [1]. These genes have deepened the knowledge of the cause of chalkiness and floury endosperm, which can be divided into the following categories: genes responsible for starch synthesis [5,6], amyloplast development [7,8], protein synthesis, storage, and transport [9,10], mitochondria function [11,12], transcription, and post-transcription [13,14]. Besides the genetic factors, environmental conditions and management approaches also significantly affect rice grain appearance, where temperature has been an important factor accompanying global warming [15]. High-temperature ripening of rice grains causes damage to endosperm starch, which leads to the deterioration in eating properties, such as the hardness and stickiness of boiled rice grains and pasting properties of rice flour.

The evaluation of CEQ is typically based on three physicochemical characteristics of starch: amylose content (AC), gel consistency (GC), and gelatinization temperature (GT) [16]. AC is the major determinant of rice eating and cooking qualities [17]. Amylose, a constituent of starch, is the primary focus in determining grain quality. Rice with high AC would become dry and flaky when cooked, whereas low AC rice would have a soft and sticky texture. Rice varieties are grouped into waxy (0–2%), very low (3–9%), low (10–19%), intermediate (20–25%), and high (>25%) AC classes based on their AC values [6]. It has been known that the granule-bound starch synthase (GBSS), encoded by the *Waxy* (*Wx*), plays a very important role in determining AC in endosperm starch [18]. So far, at least nine *Wx* alleles have been identified: *Wx^a^*, *Wx^b^*, *Wx^mq^*, *Wx^in^*, *Wx^mp^*, *Wx^op/hp^*, *Wx^lv^*, *Wx^mw^*/*Wx^la^* and null *Wx* [1].

GT is the temperature range at which the starch granules swell irreversibly in hot water and starch crystalline structures begin to melt, which determines the cooking time [19]. GT can be measured indirectly in terms of an alkali spreading value (ASV) using an alkaline or urea solution. High-GT rice requires more time to cook and leads to unacceptable texture [20]. A negative correlation between ASV and GT implies that lower GT values produce higher ASV values and better-tasting rice. QTL mapping showed that GT may be controlled by *starch synthase IIa* (*SSIIa*), located on the *ALK* locus of chromosome 6 [21]. *Wx* locus also affected GT, although GT has not yet been found to be correlated with AC [22].

The GC is a measurement of the hardening of cooked rice when cooled. GC can be classified as soft (>60 mm), adhesive (40–60 mm), or hard (<40 mm). Rice with hard GC is rough and fluffy and becomes hard and dry after cooling, while rice with soft GC is soft and elastic and still keeps soft after cooling. Usually, the larger the GC, the softer, stickier, better taste, and shinier rice [16]. As a quantitative trait, GC is suitable for QTL analysis, and its genetic basis is also complicated. So far, 22 QTLs for GC have been reported on chromosomes 1, 2, 3, 6, 7, and 10 [23]. A number of studies using different mapping populations have located the major gene for gel consistency at the *Wx* locus [24]; the SNP on exon 10 of the *Wx* gene, from C-T, is a significant factor influencing gel consistency [25]. *Wx* gene controls not only AC and also GC. ALK was also reported as a modifier gene for GC in rice [26].

Starch viscosity profiles tested by a rapid visco analyzer (RVA) have also been utilized to select desirable rice varieties with specific CEQ [27]. RVA is useful in evaluating the “degree of cooking” after processing rice into pre-cooked or puffed products [28]. Viscosity changes associated with starch gelatinization can be measured to characterize rice eating quality [29]. Starch viscosity characteristics included the following original components: peak viscosity (PV), trough viscosity (TV), and final viscosity (FV). Three secondary parameters, including breakdown (BDV), setback (SBV), and consistency (COV), were calculated based on the original data: BDV = PV − TV, SBV = FV − PV, and COV = FV − TV. Good CEQ varieties have higher BDV and lower SBV, COV, and FV [30]. Pasting temperature (PT) indicates the temperature at which viscosity begins to increase during heating, while peak time (PKT) refers to the time taken to reach PV [16]. As the RVA simulates rice cooking, the starch profile of rice is closely associated with this process and rice taste quality. It is clear that the *Wx* gene is a key determinant in the control of RVA profile parameters in rice, while the *pullulanase (PUL)* gene was shown to play an important role in the control of most of the RVA profile parameters [31].

There are significant correlations among the various sensory qualities of cooked rice. GC and ASV have negative associations with AC [32]. Lower AC values and higher GC values are typically associated with increased BDV and decreased SBV and COV [16,30,33,34]. Therefore, selecting both AC, ASV, and GC in conjunction with RVA profiles would be significant for improving the accuracy of selection in high-quality breeding programs.

Since it is challenging to directly measure the CEQ traits, currently, the only cloned major genes are *Wx* and *SSIIa* through QTL mapping. However, the phenotypes of chalkiness and floury endosperm are easily identifiable, and these traits usually affect CEQs with potentially low eating qualities [35]. Therefore, by screening for mutants with floury endosperm, it is possible to clone minor-effect genes that influence the CEQs, thus overcoming the bottleneck of relying solely on the *Wx* and *SSIIa* genes for improving rice eating quality in molecular breeding. Eight rice mutants with varying degrees of floury endosperm were used to analyze changes in CEQs under one gene mutated in each mutant. This research provides valuable genetic resources for understanding the fine structure of starch with respect to the textural properties of cooked rice grains.

## 2. Results

### 2.1. Mutants Exhibited Varying Degrees of Floury Endosperm

**Figure 1:**
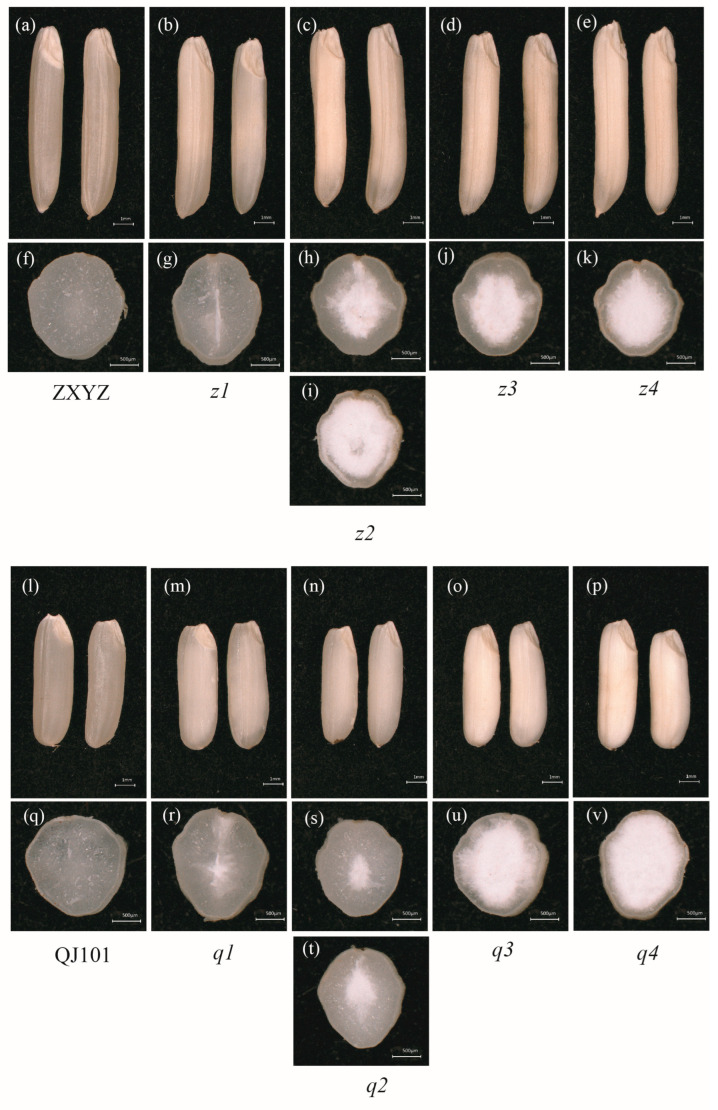
Appearance of WT and eight mutants seeds. (**a**–**e**) Comparison of ZXYZ and *z1*–*z4* seeds, Scale bars: 1 mm; (**f**–**k**) Cross-sections of ZXYZ and *z1*–*z4* seeds, Scale bars: 0.5 mm; (**l**–**p**) Comparison of QJ101 and *q1*–*q4* seeds, Scale bars: 1 mm; (**q**–**v**) Cross-sections of QJ101 and *q1*–*q4* seeds, Scale bars: 0.5 mm.

To isolate mutants defective in starch properties, M_3_ populations created by EMS mutagenesis from varieties ZXYZ (*Oryza sativa* L., *indica*) and QJ101 (*Oryza sativa* L., *indica*) were screened. Moreover, 55 independent mutants were isolated with different floury degrees (FD) endosperm. Among them, eight mutants with single gene mutations (selfed heterozygous plants showed 3:1 segregation in segregating populations, Supplementary Table S1) were further selected for investigation of their starch properties and kernel morphology. The whole surface and cross-fractured planes of wild type (ZXYZ and QJ101) and eight floury endosperm mutants were observed under a stereoscopic microscope (Figure 1).

Compared with the completely transparent endosperm of ZXYZ and QJ101 (Figure 1a,f,l,q), all eight mutants showed a floury phenotype of varying degrees. In *z1* and *q1*, a linear floury part of the central endosperm was observed (Figure 1b,g,m,r). Mature seeds from a single panicle of *z2* and *q2* mutants showed two types of FD, suggesting that starch synthesis was differently affected in these two mutants among different individuals (Figure 1c,h,i,n,s,t). Floury endosperm almost filled the whole seeds in *z3*, *z4*, *q3*, and *q4* mutants (Figure 1d,e, j,k,o,p,u,v). The selected eight mutants with different FD of endosperm represented different extents of altered starch components, suggesting different genes are involved in the process of starch synthesis in different parts of the endosperm. The starch properties of the eight mutants were investigated further in the present study.

### 2.2. Mutants Show Abnormal Development of Starch Granules and Amyloplasts

Starch, accounting for over 70% of seed weight, plays a crucial role in rice quality. The morphology of starch granules reflects the quality of rice to a great extent. In rice endosperm, insoluble starch granules (SGs) are formed in amyloplasts, which are organelles involved in starch synthesis and storage. A single amyloplast is assembled from dozens of polyhedral SGs that have sharp edges and are easily separable. SGs can be visualized easily under light microscope by staining with iodine solution.

**Figure 2:**
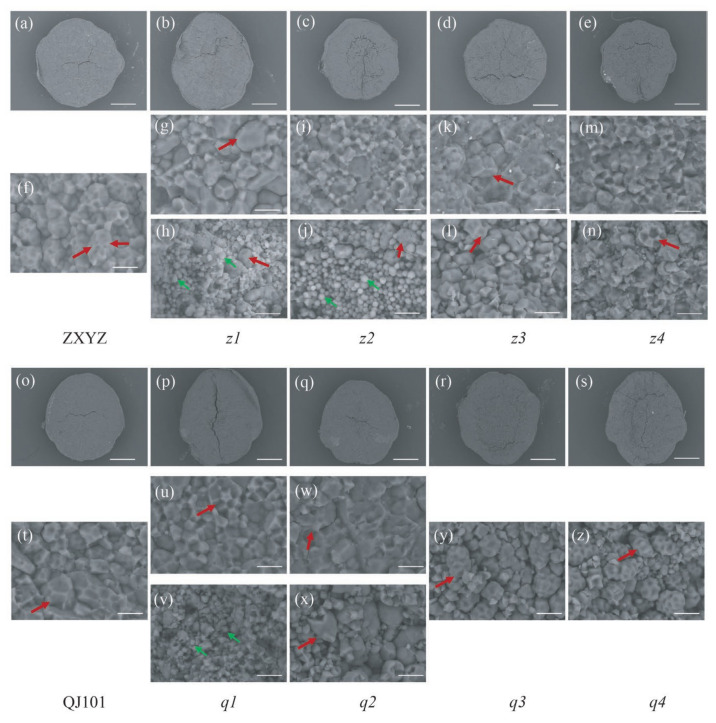
SEM analysis of the endosperm of the WT (**a**,**f**,**o**,**t**) and eight mutants (**b**–**e**,**g**–**n**,**p**–**s**,**u**–**z**). Scale bars: 0.5 mm in (**a**–**e**,**o**–**s**); 10 μm in (**f**–**n**,**t**–**z**). The compound SGs and individual SGs are outlined with red and greenarrows, respectively.

SEM was utilized to observe the morphology of compound SGs, which were tightly packed and polyhedral in the wild type and transparent part of endosperm in *z1*–*z4*, *q1*, and *q2* (Figure 2f,g,i,k,m,t,u,w). The quantity of compound SGs in *z1*–*z2* and *q1* is significantly reduced, replaced by multiple individual SGs. Also, the size of compound SGs is smaller in *q1* than in WT. Moreover, compound SGs were loosely packed and spherical in the floury parts of *z3*–*z4*, *q2*–*q4* endosperm (Figure 2l,n,x,y,z), indicating weakly filled starch in these amyloplasts.

**Figure 3:**
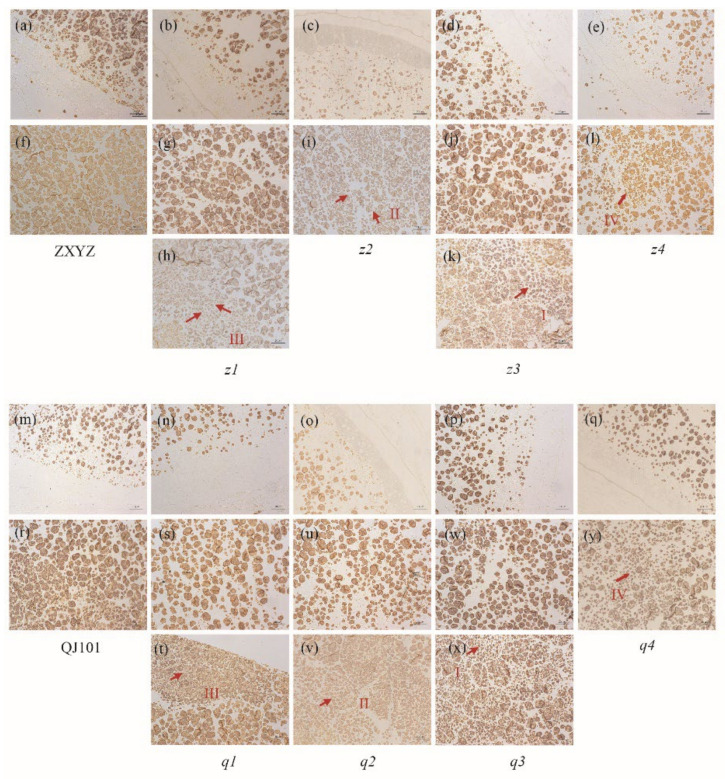
Semi-thin sections of WT (**a**,**f**,**m**,**r**) and eight mutants (**b**–**e**,**g**–**l**,**n**–**q**,**s**–**y**) endosperm at 7 days after flowering (DAF). (**a**–**e**), m-q represent the periphery of endosperm cells. (**f**–**l**,**r**–**y**) represent the center of endosperm cells, among which (**g**,**j**,**s**–**w**) indicate transparent part of corresponding mutants, (**h**,**i**,**k**,**l**,**t**,**v**,**x**,**y**) indicate floury part of corresponding mutants. Four types of single granules are labeled as I, II, III, and IV and outlined with redarrows. Scale bars: 20 μm.

**Figure 4:**
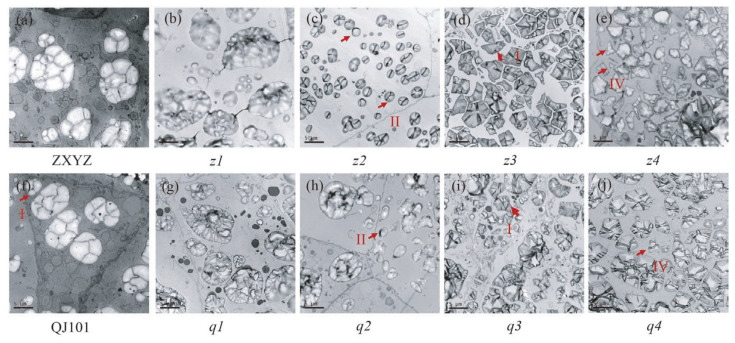
Transmission electron microscope analysis of the starch grains of WT (**a**,**f**), *z2*–*z4* (**c**–**e**), *q2*–*q4* (**h**–**j**) at 7 DAF. Floury part of *z1* (**b**) and *q1* (**g**) endosperm failed to be observed using TEM because of the difficulty in obtaining the floury section; b and g showed the normal starch grains in *z1* and *q1*. Three types of single SGs are labeled as I, II, and IV and outlined with redarrows. Scale bars: 5 μm.

TEM and semi-thin sections microscopy were prepared to observe the SG morphology of developing endosperm. In both the center and periphery of endosperm cells from wild-type, each amyloplast consists of several dozen polyhedral, sharp-edged granules, with very few single SGs present in each cell (Figure 3a,f,m,r and Figure 4a,f). Compared to the wild-type, abnormal SGs were more abundant in the floury parts of endosperm cells (Figure 3h,i,k,l,t,v,x,y and Figure 4c–e,h–j), while normal-looking SGs were enriched in the peripheral endosperm cells below the aleurone layer and non-floury parts in all mutants (Figure 3b–e,g,j,n–q,s,u,w and Figure 4b,g). Consistent with SEM characterization, most of the compound SGs were replaced by numerous single SGs in the floury parts of cells in *z1*–*z2*, *q1*, and coexisted with single SGs in *z3*–*z4*, *q2*–*q4*.

Four types of individual grains were observed in the floury parts of the eight mutants. The first type was polyhedral and sharp-edged, similar to the wild-type, indicating that some normal starch synthesis still occurred, and was observed in all mutants, such as in *z3* and *q3*, where it is likely that compound SGs broke up into single SGs but did not affect the starch morphology (Figure 3k,x and Figure 4d,i). The second type had large numbers of spherical granules compared to the polyhedral ones, as seen in *z2* and *q2*, where almost all single SGs turned round, especially in *z2* (Figure 3i,v and Figure 4c,h). The third type had large numbers of smaller granules compared to the wild type, as seen in *z1* and *q1* (Figure 3h,t). The fourth type was more sharp-edged than the wild type, as seen in *z4* and *q4*, especially in *z4*, where fragmented single SGs with irregular shapes and rough surfaces filled the cells (Figure 3l,y and Figure 4e,j). Compound SGs morphology was smaller in *z1* and *q1* (Figure 3h,t), sharp-edged and distorted in *z4* (Figure 3l and Figure 4e), and similar to relevant single SGs, but unchanged in *z2*–*z3*, *q2*–*q4* under TEM and semi-thin sections microscopy. This suggests that different genes mutated, resulting in different changes in starch morphology and structure. In the future, the cloning of mutated genes in these mutants, especially in *z2* and *z4*, two mutants with contrasting starch granule morphology, and the study of possible coordinated regulatory molecular mechanisms between genes will contribute to the improvement of the molecular mechanism of starch granule formation.

### 2.3. Mutants Showed Significant Changes of CEQs

**Figure 5:**
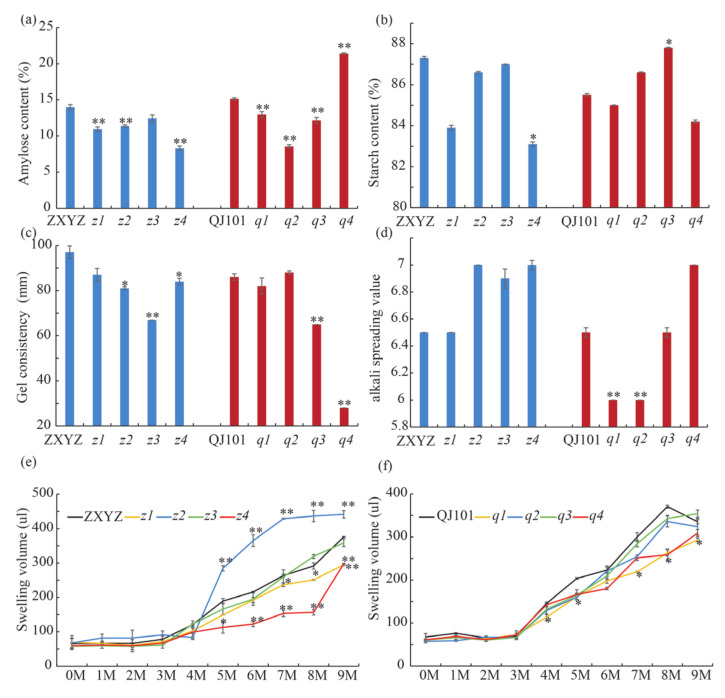
Properties and physicochemical characteristics of starches in WTs and eight mutants. (**a**–**d**) CEQs and total starch content of WTs and eight mutants. (**e**,**f**) Volume of WTs and eight mutants endosperm starch swelling in different concentrations of urea. Values are means ± SDs (n = 3). The asterisks indicate statistical significance between the wild type and the mutants, as determined by a Student’s *t*-test (* *p* < 0.05; ** *p* < 0.01).

Since AC, GC, and ASV are three major indirect indices reflecting eating and cooking quality, they were measured using milled rice flour or milled rice (Figure 5). The starch from ZXYZ and QJ101 contained about 15% AC. The *z1*, *z2*, *z4*, and the *q1*–*q3* mutants had highly significant decreases in AC, ranging from 8% to 12.9%. In contrast, the *q4* mutant showed a highly significant increase in AC, reaching 21.4% (Figure 5a). In each mutant, lower GC was observed compared to the wild-type, with a highly significant decrease in *z3*, *q3*, and *q4*. The *q4* mutant showed a 67.4% decrease in GC, indicating that the mutated gene was important for GC (Figure 5c). The ASV of the eight mutants ranged from 6 to 7, with *q1* and *q2* showing significant decreases compared to QJ101 (Figure 5d). The total starch content was dramatically decreased in *z4* when compared to ZXYZ (Figure 5b). These results demonstrate that genes mutated in the eight mutants resulted in different degrees of changes in CEQs.

### 2.4. Starch Swelling in Urea (SU) Solution Dramatically Changed in z2 and z4

Gelatinization properties of starch granules can be detected by their solubility in reagents such as urea [36]. To examine the effects of urea concentration on starch granule gelatinization in the wild type and eight mutants, we mixed endosperm starch with urea solutions ranging from 0 to 9 M (Figure 5e,f). The swelling of all starch preparations exhibited a biphasic response with increases in urea concentration starting at 5 M. However, the extent of swelling at a given concentration of urea differed among the mutants compared to the wild-type.

Compared to ZXYZ, *z2* and *z4* showed a completely opposite trend with significantly increased swelling at 5 to 9 M in *z2* but significantly decreased swelling in *z4*, suggesting a drastic change in starch structure. In the case of *z1*, the extent of swelling markedly decreased at 7 to 9 M compared to ZXYZ, with no obvious change observed in *z3*. Compared to QJ101, the extent of swelling in *q1* and *q4* markedly decreased at 4 to 9 M and 8 to 9 M, respectively, with no obvious change observed in *q2* and *q3*. These results indicate that changes in starch structure can affect the extent of swelling in urea solution.

### 2.5. Paste viscosity of Milled Rice Flour Dramatically Changed in z2 and z4

**Figure 6:**
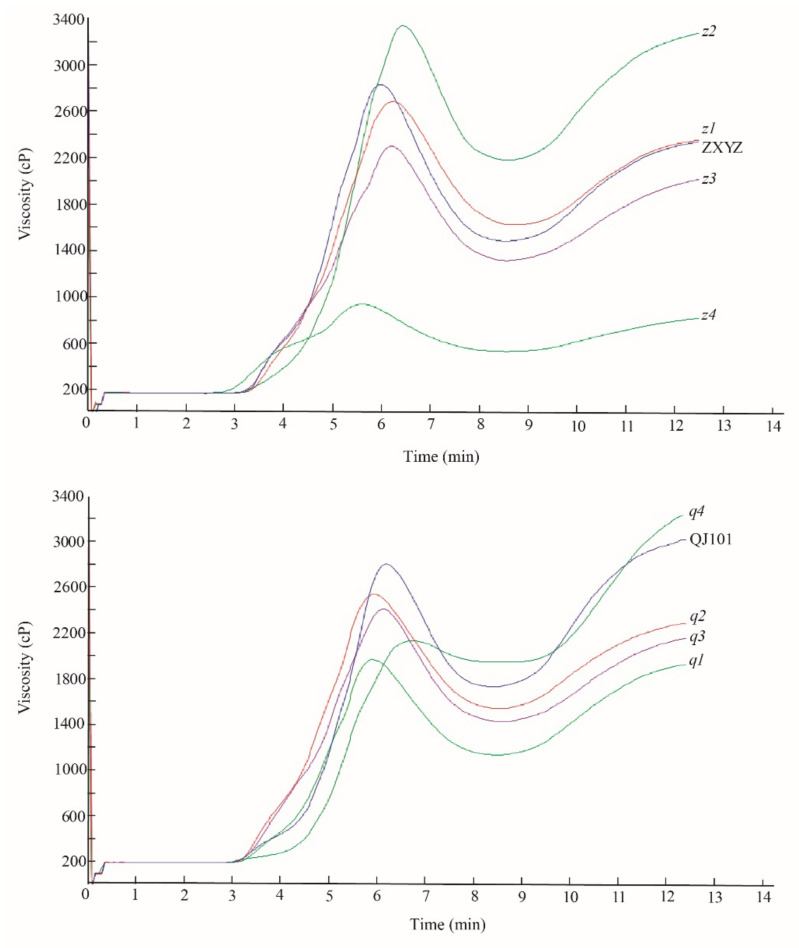
Pasting properties of endosperm starch of WTs and eight mutants.

To evaluate the effect of the eight mutations on paste viscosity, milled rice flour was analyzed using an RVA analyzer (Figure 6, Supplementary Table S2). PV was significantly decreased in all mutants compared to the wild type, except for *z2,* which showed a markedly increased PV. TV and FV were significantly reduced in *z3*–*z4*, *q1*–*q3,* and increased in *z2* and *q4*. BDV was decreased in all mutants, especially in *z4* and *q4*, resulting from a decrease of both PV and FV in *z4* and a decrease of TV and an increase of PV in *q4*. In *z2*–*z4*, the SBV was markedly increased, and in *q1*–*q3*, the SBV was significantly reduced. In the *q4* mutant, a markedly reduced PV and increased FV resulted in a significantly increased SBV. COV was decreased in *z1*, *z3*–*z4*, *q1*–*q3* and increased in *z2*. PKT was delayed in *z2* and *q4* but shortened in *z4* and *q1*–*q2*. PT was significantly elevated in *z2* and *q4*. The dramatic changes in RVA profiles of *z2*, *z4,* and *q4* indicate that the genes mutated in these three mutants are important factors controlling CEQ. The starch morphology of *z2* and *z4* has also undergone drastic changes, indicating a close relationship between starch granule formation and CEQ. This connection needs to be further explored in the future.

## 3. Discussion

### 3.1. Eight Floury Endosperm Mutants Exhibited Wide Variation in Starch Properties

In recent years, some new floury endosperm mutants were obtained through chemical or physical mutagenesis, with phenotypes similar to chalky rice, which can be regarded as extreme chalky phenotypes [37]. These mutants have altered starch properties, thereby affecting rice quality, and are valuable genetic materials for studying molecular mechanisms of rice starch synthesis and quality regulation, providing new directions for rice quality improvement. In this study, eight different floury endosperm mutants with different FD were used to analyze the changes in rice appearance quality, CEQ, and starch granule morphology compared to the wild-type. Previous studies have identified many floury endosperm mutants with different FD, including white-core mutants (*OsSSIIIa/Flo5, flo19, flo15*) [38,39,40], white belly endosperm mutants (*gif1*, *bzip58*) [41,42], floury mutants (*ssg4, gpa8, fgr1, flo16*) [7,11,43,44] and white periphery endosperm mutants (*flo7*) [45], which combined with this study indicate that starch synthesis is a complex and finely regulated process involving multiple genes. In this study, variations in FD were observed among *z2* and *q2* grains of the same panicle. It was found that this polymorphism in seed morphology remained stable across generations, suggesting it could be influenced by the environment, particularly with temperature in the grain-filling stage. This conclusion is supported by the fact that the majority of rice plants in the experiment exhibited flowering in early September, coinciding with a gradual decrease in temperatures throughout the month.

### 3.2. The Morphology of Starch Granules Affect CEQs

Rice endosperm amyloplasts produce characteristic compound-type starch granules, which consist of dozens of polyhedral, sharp-edged granules [46]. The molecular mechanisms underlying the control of starch granule morphology in cereal endosperm remain elusive, although there is evidence suggesting a possible involvement of altered membrane lipid synthesis [47]. A structural model for the compound-type amyloplast comprising an outer envelope membrane (OEM), inner envelope membrane (IEM), and intermembrane space (IMS). The IEM encapsulates each starch granule, while granules are separated by a septum-like structure (SLS) [48]. The SLS appears to function as both a stretcher, shaping amyloplasts into a spherical form, and a mold, casting starch granules into polyhedral and sharp-edged shapes [48,49]. Recent reports on floury endosperm mutants have revealed notable alterations in both amyloplasts and starch granules morphology. These mutants often exhibit rounder amyloplasts, reduced starch filling, and increased intergranular space (Figure 2, Figure 3 and Figure 4). Various oval, hemispherical, irregular starch granules have been observed (Figure 2, Figure 3 and Figure 4). However, the *z2* and *z4* mutants examined in this study exhibit two distinct phenotypes. In the *z2* mutant, nearly all starch granules have adopted a spherical shape (Figure 2j, Figure 3i, and Figure 4c), a phenotype previously observed only in the *ss3a ss4b* double mutants [50]. SS4b is important for SLS formation; the deficiency of SS3a and SS4b might weaken the SLS in that *ss3a ss4b* starch granules did not set into the mold, and starch granules and amyloplasts were not attached to their neighbors. Therefore, the starch granules remained spherical until the seed reached maturity. The crucial role of SS4b in SLS formation suggests a possible synergy or regulatory relationship between the *z2* mutated gene and *SS4b*. Conversely, *z4* mutants display a pronounced sharpening effect on both amyloplasts and starch granules (Figure 2n, Figure 3l, and Figure 4e). According to the proposed model, if SLS determines the morphology of starch granules, the extreme sharpness exhibited by the *z4* mutant may be attributed to SLS dysfunction, albeit regulated by factors with negative effects compared to those governing the *z2* mutated gene and *SS4b*. Thus, based on the opposite changes in starch granule morphology, the mutated genes of *z2* and *z4* can be named *RSGM* (round starch granule mutation) and *SRGM* (sharp starch granule mutation), respectively. Future research involving the cloning of *RSGM* and *SRGM* will deepen our understanding of this process.

Moreover, compared with the wild-type ZXYZ, SU in *z2* began to significantly increase in a 5 M urea solution while it significantly decreased in *z4*, as did the changes in PV, TV, and FV, indicating that changes in starch granule morphology can reflect changes in CEQs. It is hypothesized that the spherical shape of starch granules in *z2* may be related to the synthesis of branched chains in amylopectin. The following three reasons can explain it: (1) The urea-induced swelling of starch granules depends on the structure of branched starch rather than the extent or structure of linear starch [36]. (2) The rice gelatinization viscosity is mainly determined by linear and branched starches together [27]. (3) Spherical starch granules are possibly due to the results from lower rates of starch biosynthesis and accumulation and the significant reduction in long amylopectin chains, which connect amylopectin clusters and are important as backbones for building large amylopectin molecules in rice *ss3a ss4b* [50]. Combining the study on the *z4* mutant’s extremely pointed starch granule morphology and the opposite trends of SU, PV, TV, and FV compared to the *z2* mutant can further enhance the understanding of the relationship between starch granule morphology and the length of amylopectin branches. It should be noted that some floury mutants have changed starch granule morphology, but compared with the wild type, there is no significant difference in starch properties [7,46]. Therefore, future research should pay attention to the relationship between starch morphology and CEQs.

### 3.3. Certain Genes but May Not PUL Affect CEQs in These Floury Mutants

**Figure 7:**
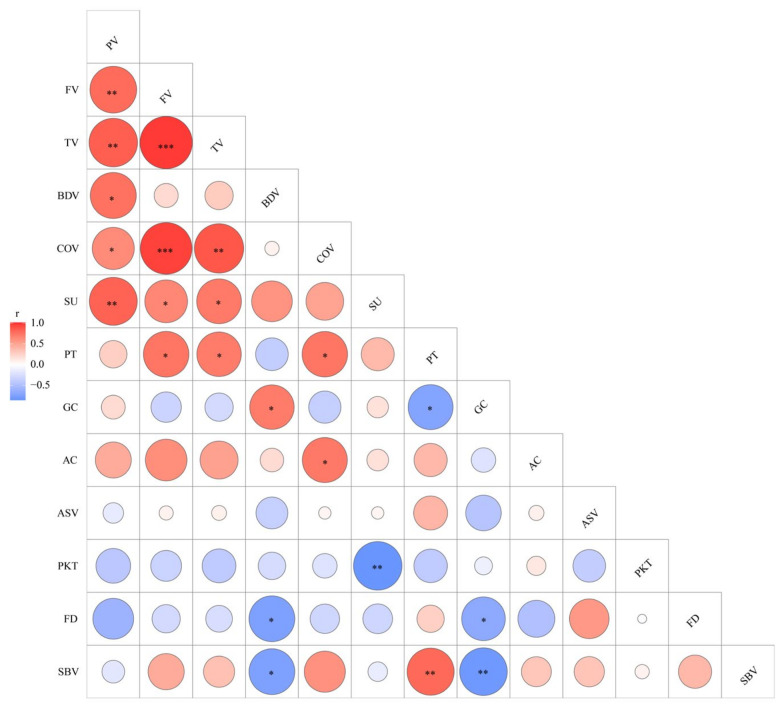
Correlation relationships of starch properties in WTs and eight mutants. The areas of roundness showed the absolute value of corresponding r, and the red and blue color of roundness indicated positive and negative correlations, respectively. The asterisks indicate statistical significance between the starch properties, * *p* < 0.05; ** *p* < 0.01; *** *p* < 0.001.

Although the eight floury mutants and their wild-type contained the same allele of *Wx* and *ALK*, they had varied AC, GC, and ASV, and these parameters showed no correlation among the mutants (Figure 7). These results demonstrated that mutations in such genes might lead to an imbalance in the correlation between GC, ASV, and AC. GC, ASV, and AC often exhibit different values under the same combination of *Wx* and *ALK* alleles, indicating that minor effect genes also play an important role in regulating GC, ASV, and AC. On the other hand, due to the use of narrow germplasm resources in a single experiment, it is often difficult to isolate genes with small effects. Therefore, by cloning the single genes mutated in some mutants that affect GC, ASV, and AC, the regulatory network of GC, ASV, and AC can be more detailed and completed.

Previous studies showed that close correlations exist between AC, GC, and RVA profiles, particularly with BDV, SBV, and COV [30,51]. However, the correlation between ASV and RVA curves is still controversial. One study using 114 varieties of japonica, indica, and glutinous rice found no significant correlation between RVA values and ASV [33], while others reported a significant correlation between ASV and RVA parameters, such as BDV, SBV, and PT [6,52]. It is clear that the choice of varieties and environmental factors, such as different planting regions and temperature, can greatly affect the results. This study found that even under single-gene control, GC was positively correlated with BDV and negatively correlated with SBV and PT, and AC was positively correlated with COV, indicating that significant correlations still exist between AC, GC, and some RVA indicators (Figure 7). Since *Wx* is the major gene controlling AC, GC, and RVA parameters, it proved that the mutated genes in the eight mutants do not include *Wx*, which is why they still exhibit stable correlations. There is no significant correlation between ASV and other indicators (Figure 7). Since previous studies using natural populations have failed to fully explain the association between ASV and other quality traits, it remains challenging to elucidate the molecular mechanisms regulating GT using a limited number of mutant materials in this study. Significant changes in ASV were observed in *q1* and *q2* mutants, and the mutated genes of these two mutants can be named *ASV-1* and *ASV-2,* respectively. Cloning *ASV-1* and *ASV-2*, without considering environmental factors, can break the current situation of only discovering SSIIa’s regulation of GT and improve the understanding of the association between ASV and other quality traits. Additionally, among the RVA profiles, a positive correlation was observed between PV, TV, and FV, while a negative correlation was found between BDV and SBV. PT was positively correlated with FV, TV, SBV, and COV, which is consistent with previous observations (Figure 7) [21,30]. With the exception of the *Wx* gene, the main gene controlling RVA parameters is *PUL*. It is speculated that these mutants retain these stable correlations among RVA parameters because they may not have any mutations in the *PUL* gene. Cloning these genes in the future will confirm the validity of this hypothesis.

In the future, cloning these mutant genes, especially the mutant genes from *z2*, *z4*, and *q4* that cause significant changes in CEQs, will contribute to the exploration of rice CEQs-related genes other than *Wx* and *SSIIa*. This will be of great significance for molecular marker-assisted breeding for rice quality improvement.

## 4. Materials and Methods

### 4.1. Plant Materials

The floury endosperm rice mutants were derived from the indica rice (*Oryza sativa* L.) varieties QJ101 and ZXYZ through EMS mutagenesis. These mutants were homozygous and self-pollinated for three generations. The wild-type rice and mutants were planted simultaneously in an experimental field at the Zhejiang Academy of Agricultural Sciences in Hangzhou, China. The mature grains were dehulled, and the brown and milled kernels were used as plant materials for this study.

### 4.2. Microscopy

Dehulled brown rice kernels and cross sections of brown rice kernels from the mid-region were photographed using a camera (VHX 950F, KEYENCE, Tokyo, Japan). Mature rice seeds were transversely cut using a knife, and samples were examined with a HITACHI TM3000 scanning electron microscope.

Transverse sections of 7 DAF endosperms (approximately 1 mm thickness) of the wild type and eight mutants were fixed overnight in 0.1 M phosphate buffer (pH 7.2) with 2% (*v*/*v*) glutaraldehyde and 2% (*w*/*v*) paraformaldehyde. After dehydration in an ethanol series, samples were embedded in LR White resin (London Resin, Berkshire, UK), sectioned using an ultramicrotome (CM1950, Leica, Wetzlar, Germany), and observed under a microscope (H7650, Hitachi, Tokyo, Japan).

Semi-thin sections of 7 DAF endosperm from the wild type and eight mutants were prepared as described by [53]. Sections (1 μm) were stained with I_2_-KI for 5 s and subsequently examined under a light microscope (Axio Vert.A1, Zeiss, Oberkochen, Germany).

### 4.3. Determination of RVA Profiles

The pasting properties of rice flour samples were measured using an RVA analyzer (Perten, Sydney, Australia) within a short period of 12.5 min. Approximately 3 g of flour (12% moisture basis) from each rice sample was weighed directly into the aluminum canister and mixed with 25 g of distilled water. The RVA dispersed the samples by rotating the paddle at 960 rpm for the first 10 s of the test, after which the viscosity was sensed using a constant paddle rotation speed of 160 rpm. The idle temperature was set at 50 °C, and the following 12 min test profiles were run in this order: (1) hold at 50 °C for 1.0 min, (2) linearly raise temperature to 93 °C until 5.5 min, (3) hold at 93 °C until 7 min, (4) linearly reduce temperature to 50 °C at 11 min, and (5) hold at 50 °C until the full 12.5 min. All analyses were conducted in triplicate. Viscosity values were recorded in centipose (cp).

### 4.4. Determination of CEQ

The starch content of rice flour was measured using a starch assay kit (Megazyme, Wicklow, Ireland, http://www.megazyme.com/, accessed on 6 February 2022) according to the manufacturer’s protocol. The amylose content was assessed following the method described by [53]. The GT was determined using the alkali digestion test. A duplicate set of six whole-milled kernels without cracks was selected and placed in a plastic box (5 × 5 × 2.5 cm). Then, 10 mL of 1.7% KOH solution was added. The samples were arranged to provide enough space between kernels to allow for spreading. The boxes were covered and incubated for 23 h in a 30 °C oven. The starchy endosperm was visually rated based on a seven-point numerical spreading scale, which serves as a standard evaluation system for rice. Based on the ASV score, the GT of rice grains can be classified into four groups: high (1–2), high-intermediate (3), intermediate (4–5), and low (6–7). GC was measured using flour (100 mg) from all samples, weighed in duplicate into 13 mm × 100 mm tubes, and 200 μL of ethyl alcohol (95%), containing 0.025% thymol blue, was added to each tube along with 2 mL of 1 M KOH. The tubes were then placed in a vigorously boiling water bath for 8 min. After the tubes were removed from the water bath, they were held at room temperature for 5 min and then cooled in an ice water bath for 20 min. Following this, the tubes were laid horizontally on a lightbox on top of graphing paper, and after 1 h, the distance that the gel migrated in the tube was measured.

### 4.5. Determination of Starch Swelling in Urea Solution

A total of 20 mg of WT and eight mutants flour was mixed with 1 mL of a solution of 0 to 9 M urea, the pH of which was adjusted to 6.0 with acetic acid in an Eppendorf tube. The mixture was incubated at 25 °C for 24 h. The suspension was centrifuged for 20 min at 8000× *g* at room temperature and then allowed to stand for 1 h. The solubility of the granules in the urea solution was expressed in terms of the volume of the swollen sediment. The volume was calculated by subtracting the volume of the supernatant from the urea solution (1 mL).

## Data Availability

Not applicable.

## Supplementary Materials

The following supporting information can be downloaded at: https://www.mdpi.com/article/10.3390/plants12203541/s1, Table S1: χ^2^ test of eight mutants; Table S2: Pasting properties of endosperm starch of WTs and eight mutants.
