# Supplementary material for: Starch Properties and Morphology of Eight Floury Endosperm Mutants in Rice
Source: Plants (Basel). 2023 Oct 12;12(20):3541. doi: 10.3390/plants12203541 (PMC10610063; doi:10.3390/plants12203541)
Supplement: Supplementary file 1 [file plants-12-03541-s001.zip › plants-2606033-supplementary.pdf]

Table S1. $\chi2$ test of eight floury mutants.

	$\chi2$	$\chi2_{0.05, 1}$
z1	1.7633333	3.84
z2	1.47	3.84
z3	0.27	3.84
z4	2.43	3.84
q1	2.8033333	3.84
q2	0.4033333	3.84
q3	0.5633333	3.84
q4	0.4033333	3.84

Table S2. Pasting properties of endosperm starch of WTs and eight mutants.

	PV (cP)	TV (cP)	FV (cP)	BDV (cP)	SBV (cP)	COV (cP)	PKT (min)	PT(°C)
**ZXYZ**	2853 ± 9.9	1511.5 ± 0.7	2362 ± 5.7	1341.5 ± 9.2	‘-491 ± 4.2	850.5 ± 4.9	6 ± 0	77.2 ± 0.6
z1	2671 ± 65.1	1601.5 ± 70	2333 ± 69.3	1069.5 ± 4.9**	’-338 ± 4.2**	731.5 ± 0.7**	6.17 ± 0.1	78.325 ± 0
z2	3329 ± 17**	2105 ± 4.2**	3193 ± 4.2**	1224 ± 12.7**	‘-136 ± 12.7**	1088 ± 0**	6.365 ± 0**	85.825 ± 0.5**
z3	2333 ± 2.8**	1335.5 ± 10.6**	2020.5 ± 24.7**	997.5 ± 13.4**	’-312.5 ± 27.6*	685 ± 14.1**	6.135 ± 0.1	77.15 ± 0.6
z4	963.5 ± 6.4**	554.5 ± 6.4**	839 ± 7**	409 ± 0**	‘-124.5 ± 0.7**	284.5 ± 0.7**	5.565 ± 0**	74.875 ± 0.5
**QJ101**	2842.5 ± 24.7	1757 ± 7	3022 ± 7	1085.5 ± 31.8	’179.5 ± 17.7	1265 ± 14.1**	6.27 ± 0	78.725 ± 0.6
q1	2006.5 ± 19**	1165 ± 22.6**	1965 ± 26.9**	841.5 ± 3.5**	‘-41.5 ± 7.8**	800 ± 4.2**	5.93 ± 0**	79.15 ± 0
q2	2608.5 ± 58.7*	1586.5 ± 36*	2346 ± 46.7**	1022 ± 22.6	’-262.5 ± 12**	759.5 ± 10.6**	5.965 ± 0*	76.825 ± 0
q3	2425.5 ± 12**	1443 ± 4.2**	2168 ± 11.3**	982.5 ± 7.8*	‘-257.5 ± 0.7**	725 ± 7**	6.165 ± 0	77.925 ± 0.6
q4	2143 ± 22.6**	1966 ± 8.5**	3241.5 ± 38.9*	177 ± 14.1**	’1098.5 ± 16.3**	1275.5 ± 30.4	6.835 ± 0**	90.95 ± 0**

The asterisks indicate statistical significance between WTs and eight floury mutants, *P < 0.05; **P < 0.01.
